# Reduction of Toxic Metal Ions and Production of Bioelectricity through Microbial Fuel Cells Using *Bacillus marisflavi* as a Biocatalyst

**DOI:** 10.3390/molecules29122725

**Published:** 2024-06-07

**Authors:** Rojas-Flores Segundo, Magaly De La Cruz-Noriega, Cabanillas-Chirinos Luis, Nélida Milly Otiniano, Nancy Soto-Deza, Walter Rojas-Villacorta, Mayra De La Cruz-Cerquin

**Affiliations:** 1 Instituto de Investigación en Ciencias y Tecnología de la Universidad Cesar Vallejo, Trujillo 13001, Peru; mdelacruzn@ucv.edu.pe (M.D.L.C.-N.); notiniano@ucv.edu.pe (N.M.O.); nsoto@ucv.edu.pe (N.S.-D.); mdelacruz@ucv.edu.pe (M.D.L.C.-C.); 2 Investigación Formativa e Integridad Científica, Universidad César Vallejo, Trujillo 13001, Peru; lcabanillas@ucv.edu.pe (C.-C.L.); wrojasv@ucv.edu.pe (W.R.-V.)

**Keywords:** agricultural wastewater, heavy metals, removal, bioelectricity, microbial fuel cells

## Abstract

Industrialization has brought many environmental problems since its expansion, including heavy metal contamination in water used for agricultural irrigation. This research uses microbial fuel cell technology to generate bioelectricity and remove arsenic, copper, and iron, using contaminated agricultural water as a substrate and *Bacillus marisflavi* as a biocatalyst. The results obtained for electrical potential and current were 0.798 V and 3.519 mA, respectively, on the sixth day of operation and the pH value was 6.54 with an EC equal to 198.72 mS/cm, with a removal of 99.08, 56.08, and 91.39% of the concentrations of As, Cu, and Fe, respectively, obtained in 72 h. Likewise, total nitrogen concentrations, organic carbon, loss on ignition, dissolved organic carbon, and chemical oxygen demand were reduced by 69.047, 86.922, 85.378, 88.458, and 90.771%, respectively. At the same time, the PD_MAX_ shown was 376.20 ± 15.478 mW/cm^2^, with a calculated internal resistance of 42.550 ± 12.353 Ω. This technique presents an essential advance in overcoming existing technical barriers because the engineered microbial fuel cells are accessible and scalable. It will generate important value by naturally reducing toxic metals and electrical energy, producing electric currents in a sustainable and affordable way.

## 1. Introduction

Globalization has allowed industries to grow at an accelerated rate, increasing the amount of industrial wastewater. Many industries use heavy metals to manufacture their products, causing the wastewater discharges to contain high amounts of these metals [1,2,3]. When wastewaters with a high content of soluble heavy metals mix with the aquatic environment, it significantly threatens the living organisms present and the people who use these waters [4]. The literature shows that the most frequently contaminating metals are copper (Cu), arsenic (As), lead (Pb), zinc (Zn), titanium (Ti), and platinum (Pt) [5,6].

Among heavy metals, arsenic is a toxic contaminant for living beings because it damages the respiratory, digestive, and circulatory systems and is associated with lung, liver, and skin cancers. For this reason, its elimination or reduction in wastewater has been attempted by several techniques, for example, treatment by coagulation, sorption, or separation by membranes, which are generally used for low concentrations of arsenic (up to 30 µmol L^−1^) [7,8,9]. However, microbial fuel cells (MFCs) have recently begun to lead in the bioremediation of water contaminated with heavy metals [10]. MFCs use any substrate as fuel for generating electricity, which is converted into electrical energy from the redox reactions within the chambers (anodic and cathodic) that compose them [11,12,13]. However, the electrodes that compose them have been capable of absorbing different types of heavy metals, and the use of microorganisms has also helped reduce the concentrations of these metals [14,15]. For example, it has been shown that *Rhizobacteria* in MFCs can reduce the high contents of toxic metal ions such as Pb, Cr, Cd, As, and Cr from 100 mgL^−1^ by 23.86% [16]. Likewise, the Gram-negative soil bacterium *Rhizobium anhuiense* has been studied in MFCs, reporting a generated voltage of 635 mV in 2.59 mWm^−2^ of power density [17]. Zhao et al. (2021) used the *Corynebacterium* vitaeruminis LZU47-1 strain in a dual-chamber MFC using a biocathode and reduced the Cd concentration by 98.63%, generating a voltage of 120 mV, 252.36 mWm^−2^ of power density, and 102.5 Ω of internal resistance [18].

In Peru, there is informality among companies whose wastewater has a high content of heavy metals that needs to be adequately treated before being dumped [19]. For example, illegal mining in mountain areas has increased in recent years, and the metals used in the extraction are thrown directly into rivers. Farmers use the water of rivers to irrigate their fields, thus seriously damaging the crops of fruits and vegetables irrigated with these waters [20,21]. For example, Zapana et al. (2020) studied wastewater from the city of Arequipa, Peru, and found a high content (250 mg/L) of chromium [22]. Likewise, Cáceda et al. (2023) found a high cyanide (CN) content in the mining soils of the city of Tacna, Peru, in which bacteria were used for bioremediation [23]. In this sense, high contents of heavy metals have also been found in plants, fruits, and vegetables irrigated with contaminated water. Schiller et al. (2020), in their research, found Hg, Pb, Cd, and Cr in crops in which, when ingested by people, these metals disperse in the organs of the human body, causing blood cancer in a certain number of people [24]. Castro et al. (2023) found Pb, Cd, and As in milk and its derivatives in their research. The milk came from cattle whose drinking water was from a nearby river, and sewage from illegal mining companies had been dumped into this river for years [25]. Furthermore, single-chamber MFCs tend to obtain better anodic biofilm formation because the bacteria show nanowires with connections spread throughout the cell fibers [18]. The biofilm formation helps improve the passage of electrons from the medium (substrate) to the electrode by acting as a mediator. Likewise, electrodes made of carbon or carbon itself have been the most used in the literature [10,13]. These electrodes have been manufactured with metallic materials because their metallic nature favors the transport of electrons by reducing their internal resistance to the MFC [18]. The use of agricultural water contaminated with toxic metal ions as a substrate in a single-chamber MFC and *Bacillus marisflavi* as a biocatalyst has yet to be reported in the literature; the scientific gap in this field must be covered. The research question is as follows: Could *Bacillus marisflavi* be a biocatalyst to reduce toxic metals such as arsenic, copper, and iron and generate bioelectricity simultaneously? Its hypothesis is the following: Yes, because its biotechnological potential has been observed*; B. marisflavi* reduces toxic metals through biosorption processes and has the bioelectric potential to generate electrons because the Bacillus genus can produce extracellular polymeric substances (EPSs) that adhere to the anode, making electron transport possible.

The aim of this research is to explore the potential of *Bacillus marisflavi* as a microbial fuel cell biocatalyst, capable of generating electricity and reducing toxic metal ions (Fe, Cu, and As) in the irrigation water of the Moche River, Trujillo, Peru. Over a twelve-day period, we measured voltage using a digital multimeter, internal resistance using a potentiostat, electrical conductivity (EC) using a conductivity meter, current using an ammeter, PD vs. CD using a voltammetry technique, dissolved organic carbon (DOC) using a spectrophotometer, total organic carbon using a carbon analyzer, loss on ignition (LOI) using a muffle furnace, total nitrogen (TN) using the Kjeldahl method, and chemical oxygen demand (COD) using a COD analyzer. The results of this study will be the first from a prototype using this type of bacillus on a 120-L scale, operating at room temperature. Two MFCs were employed in this investigation, one as a control (containing only the substrate and the electrodes) and another with the *Bacillus marisflavi* bacteria (containing the substrate, electrodes, and *Bacillus marisflavi*), to assess the potential positive effects of microorganisms in the generation of electrical energy and reduction in toxic metals. This research holds promise for mining, tanning, agro-industrial, and other companies, offering a scalable technology that can effectively eliminate or reduce heavy metals to permissible levels while simultaneously generating environmentally sustainable electrical energy.

## 2. Results and Discussion

**Figure 1:**
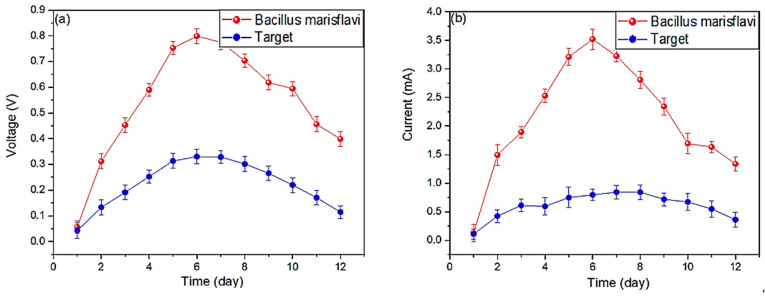
Report of the generated measurements of (**a**) electric potential and (**b**) electric current.

The initial voltage values were practically equal, being 0.056 and 0.042 V in the MFC-SC with *Bacillus marisflavi* and the MFC-Target, respectively; these values increased until the sixth day, where the MFC–*Bacillus marisflavi* (0.798 V) showed a 58.62% higher voltage than the MFC-Target (0.3305 V), as shown in Figure 1a. This phenomenon of increasing and decreasing voltage values was also observed by Sakr et al. (2023), who used *Bacillus piscis* as a biocatalyst in their MFCs and mentioned that use life of their wastewater limited the potential of each MFC. This potential can be regenerated by introducing a new substrate [26,27]. The same phenomenon was also observed by Ren et al. (2021) who used Saccharomyces cerevisiae and *Pseudomonas aeruginosa*; the potential generated was limited by the redox processes originating in the chambers of the MFCs, which, due to not having more useful substances for realization after this process, began to show decreasing values [28]. According to Sabri et al. (2021), the useful life of each substrate is related to the carbon sources present in the substrate and the growth rate of the microorganisms used within the MFCs [29]. Dongre et al. (2022) demonstrated that electrical potential values can increase using low-frequency ultrasound because it increases the bacterial growth rate as the cell membranes are altered in some way, which facilitates membrane selectivity, improving the transport of molecules [30]. Treesubsun C. and Thiravetyan P. (2021) mentioned that a low oxygen concentration in the anodic chamber directly affects the electrical production potential due to the very nature of the microorganisms used. They also mentioned that for MFCs using certain types of microorganisms, it must be standardized to obtain the maximum potential in each MFC [31]. The values of the electric current in this study are shown in Figure 1b, where the values on day 1 were 0.13 and 0.11 mA for the MFC–*Bacillus marisflavi* and the MFC-Target, respectively; these values increased until day six, where the MFC–*Bacillus marisflavi* (3.519 mA) showed a 342.05% higher value than the MFC-Target (0.796 mA), and then gradually decreased until the last day. The electrical current values increased in the first days due to the formation of biofilms and the capture of electrons by the electrodes in the metabolism process of the microorganisms present in the MFCs [32,33]. The MFC–*Bacillus marisflavi* had higher electrical current values possibly because the *bacillus* used helped the good formation of the anodic biofilm [34]; this phenomenon was observed in other investigations as well. Victoria A. and Mercy. F. (2021) mention in their research that the microorganisms attached to the electrodes helped generate electrons. However, as the mortality rate of the bacteria increased and the nutrients showed up towards the last day, the electric current values decreased [35]. It is essential to know the effect of pH on the production of electrons in MFCs and to identify the initial microorganisms in MFCs and adequately standardize this parameter because microorganisms grow at appropriate pH, which would impair efficiency [36].

The total nitrogen values decreased by 69.047 and 66.6% for the MFC–*Bacillus marisflavi* (0.52 mg/L) and the MFC-Target (0.56 mg/L), respectively, compared to their initial value (1.68 mg/L), as shown in Table 1. This value is considerably high compared to other investigations. However, some investigations that introduced glucose to the substrate managed to increase the percentage of total nitrogen removal; for example, Tao et al. (2020) managed to reduce it by 87.66 ± 4.23% by introducing 60% glucose in the substrate used for their single-chamber MFCs [37]. Some reports mention that denitrification increases enormously with more excellent activity of heterotrophic bacteria in wastewater used as a substrate [38]. Ge et al. (2020) managed to reduce the amount of nitrogen present in their MFC substrate by 62.4 ± 2.2% due to mixotrophic denitrification (heterotrophic and autotrophic), which occurs when the carbon source amount is high, but if the carbon source amount is insufficient or low, heterotrophic denitrification weakens and autotrophic denitrification dominates [39]. Here, the values of total organic carbon decreased by 86.922 and 79.926% for the MFC–*Bacillus marisflavi* (50.1 mg/L) and MFC-Target (76.9 mg/L), respectively, contrasted with the initial concentration (383.1 mg/L); these values are high compared to other research, as seen in Table 1. Ye et al. (2020) managed to reduce the total organic carbon by 45% using wastewater from animal corpses in their MFC as a substrate, mentioning that the design of the MFC and the amount of substrate that enters the anodic chamber can help reduce this parameter if microbes are efficient in the degradation process [40]. Bacteria use organic carbon to produce CO_2_ and electrons, so an optimal growth rate in the MFC generates more excellent organic carbon removal. Stimulating the oxidation process of organic matter improves the removal efficiency of total organic carbon [41,42]. The loss on ignition (LOI) showed a decrease in value by 85.378 and 79.895% for the MFC–*Bacillus marisflavi* (16.8 mg/L) and MFC-Target (23.1 mg/L), respectively, contrasted with the initial concentration (114.9 mg/L). The MFC microorganism *Bacillus marisflavi* had better adhesion to the anode, making the soluble carbon concentration an essential factor for the performance of the MFC [43]. Thus, the fermentative products improve the diversity in the substrate, making exoelectrogenic bacteria a rich source of nutrients for their reproduction and making the MFC more efficient [44]. The dissolved organic carbon (DOC) values decreased by 88.458 and 79.934% for the MFC–*Bacillus marisflavi* (28.3mg/L) and MFC-Target (49.2 mg/L), respectively, compared to their initial value (245.2 mg/L) [45]. This high decrease is due to the organic matter in the degraded substrate used to restrain the microbes that generated the bioelectricity [46]. According to Liang et al. (2021), a decrease in DOC values harms the current density performance because a decrease in compounds such as acetate, glucose, and carboxylic acids would cause the poor performance of microorganisms in MFCs [47]. The chemical oxygen demand (COD) showed a decrease in its values by 90.771 and 86.469% for the MFC–*Bacillus marisflavi* (38.4 mgO_2_/L) and MFC-Target (56.3 mgO_2_/L), respectively, compared to the initial value (416.1 mgO2 /L). Marassi et al. (2020) managed to eliminate 86% of COD using dairy wastewater and a consortium of *Shewanella* oneidensis as a substrate, mentioning that this was due to the increase in bio-electrochemical oxidation, which caused a more significant discharge of electrons towards the external circuit, resulting in greater consumption of COD [48]. Likewise, Petel et al. (2021) managed to generate a removal of 90 ± 1.5% using textile wastewater and *Exiguobacterium* sp. as the substrate in an MFC, mentioning that the use of dye-degrading bacteria in activated sludge in the wetland macrocosm considerably improved the reduction in COD [49]. Sun et al. (2020) mentioned that incomplete degradation with compounds that are more difficult to biodegrade and oxidize will decrease the reduction in COD and, therefore, decrease the energy efficiency [50].

The following concentrations of As, Cu, and Fe were obtained during the monitoring of the MFCs: the arsenic concentration reduced by 99.08% for both the MFC–*Bacillus marisflavi* and MFC-Target, while decreases of 56.08 and 14.53% in copper values and decreases of 91.39 and 84.63% in iron values were observed for the MFC–*Bacillus marisflavi* and MFC-Target, respectively, all in 72 h (Table 2). Spontaneous oxidation processes are an essential factor for the reduction in arsenic particles, and mixed cultures help the adsorption of arsenic present on substrates and the faster transfer of electrons to the anode electrode [50]. San-Martin et al. (2023) recently reduced the arsenic concentration in wastewater by 100% through cells using *Bacteroides*, *Chloroflexi*, and *Firmicutes* as biocatalysts [51]. Sobhani et al. (2023) reduced the amount of copper in their wastewater by 50% with a pH 3 operation of their MFC, mentioning that anaerobic operation stimulates the adhesion of Cu particles to the graphite electrode used in microorganisms [52]. Wu et al. (2018) managed to remove 78.8% of Cu in an MFC when using sludge as a substrate, mentioning that the decrease in Cu was due to the reductive reactions in the oxidation process of organic compounds, where the substrate was oxidized by the microorganisms electrochemically in order to produce electrons and protons [53]. Gonzalez et al. (2022) reduced the percentage of iron by 60% using sludge as a substrate in microbial fuel cells, mentioning that this reduction was due to the presence of planktonic cells [54]. Becerril et al. (2021) achieved iron reductions in the range of 80–90% in their MFC when using wastewater as a substrate, mentioning that the acetate present in their samples was used by the exoelectrogenic bacteria as a source of preferred carbon [55].

**Figure 2:**
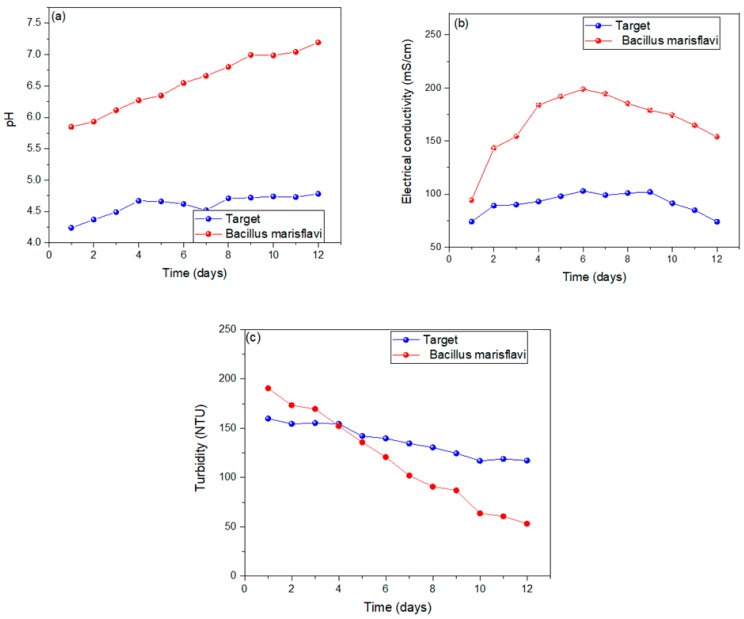
Report of the monitoring carried out of (**a**) pH, (**b**) electrical conductivity and (**c**) turbidity.

The pH concentrations were monitored for twelve days of operation of the MFCs, demonstrating that the MFC–*Bacillus marisflavi* operated in a neutral regime and the MFC-Target operated in a moderately acidic regime, with their optimal operating pH being 6.54 and 4.62, respectively, as shown in Figure 2a. The optimal operating pH reported here is similar to other investigations where the optimal pH was also around 6; for example, Attia et al. (2024) investigated wastewater in an MFC with an optimal pH of 6.60, mentioning that the durability of the existing microbes in the MFC-SC was due to the optimal standardization of their pH values [56]. Likewise, Li et al. (2021) operated a substrate used at a pH of 8.5, mentioning that the high voltage values reported were because the bacterial communities operated at optimal conditions, managing to form anodic and cathodic biofilms [57]. Figure 2b reports that the monitored concentrations of electrical conductivity of the substrates used rose from first day until the sixth day, with values of 198.72 and 103.18 mS/cm for the MFC–*Bacillus marisflavi* and MFC-Target, respectively, and then fell until the last day, on which the values were 153.83 and 73.96 mS/cm for the MFC–*Bacillus marisflavi* and MFC-Target, respectively. The values increased because in the first days of operation, the microorganisms decomposed the organic components, causing the electrons to be released; as the organic matter of the substrate decreased, the substrate became more resistant to passage. The transport of electrons was causing the values of electrical conductivity to decrease [58,59]. Figure 2c shows the turbidity concentrations observed during the monitoring, showing a decrease from the first day with values of 190.58 and 159.77 NTU to 53.19 and 117.31 NTU on the last day for the MFC–*Bacillus marisflavi* and MFC-Target, respectively. A decrease in contaminants, particulate matter sedimentation, and organic matter degradation causes a reduction in turbidity values. Some investigations reported decreases in these values and related it to the decontamination of wastewater [60,61]. Hs et al. (2020) reduced the turbidity of wastewater used as a substrate in their MFCs by 88.05% in 240 h of operation [62]. Rossi et al. (2022) reported that the turbidity values in their MFC increased by 78%, mentioning that the reduction in this parameter was due to the dispersion of the solids and particles of the substrate that were effectively eliminated in the MFC treatment process [63].

**Figure 3:**
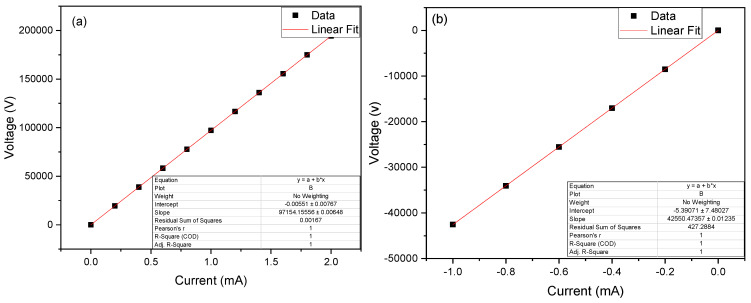
Report of calculated internal resistance values for the MFCs used: (**a**) Target, and (**b**) with *Bacillus marisflavi*.

**Figure 4:**
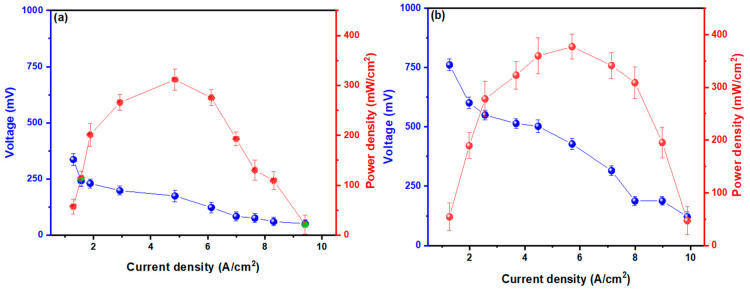
Report of the calculations carried out for PD vs. CD for the MFCs used: (**a**) Target and (**b**) with *Bacillus marisflavi*.

The internal resistance values can be observed in Figure 3, which were calculated using Ohm’s law (V = IR), where the value of electric current is in the “x” axis and the electric potential is in the “y” axis. Through a linear adjustment, the value of the resulting slope is the Rint. These values for the MFC–*Bacillus marisflavi* and MFC-Target were calculated as 42.550 ± 12.353 and 97.154 ± 6.480 Ω, respectively. The low reported values are due to the intrinsic characteristic of the electrodes used, which, being metallic, facilitated the passage of electrons [64]. Rahdar et al. (2024) obtained a Rint. of 446 Ω and mentioned that this was due to the poor formation of biofilms (anodic and cathodic), which did not allow electrons to pass through the external circuit of the cell [65]. Tamilarasan et al. (2024) mentioned in their research that the size and selection of the electrode used have an effect on the formation of biofilms by microbes, and that the Rint. of MFCs can be reduced by electrode selection, reactor design, electrode spacing, and wastewater inlet rate [66]. PD and CD measurements are shown in Figure 4; the MFC–*Bacillus marisflavi* and MFC-Target showed a DPmax of 376.20 ± 15.478 and 310.559 ± 16.471 mW/cm^2^ at a DC of 5.713 and 4.917 A/cm^2^ with peak voltages of 760.620 ± 26.491 and 333.898 ± 22.582 mV, respectively. In their research, Ghasemi M. and Rezk H. (2024) reported that PD improves by introducing an amount of carbon and nitrogen and an aeration rate to the substrate [67]. In this sense, Arun et al. (2024) mentioned in their research that the PD values can increase if the measurements of the Rint. of the MFC decrease, and this can be achieved by varying the space between the electrodes and avoiding the use of catholyte for the connections between the cameras [68]. Yang et al. (2024) reported a PD of 430 mW/m^2^ using activated sludge (substrate), indicating that the electrode made of carbon nanotubes improved the generation of PD by 35% compared to that made of graphite [69]. Likewise, Surti et al. (2024) reported a PD of 407.23 mW/m^2^, mentioning that the PD can be improved if the R_ext_ of the cell is equivalent to the Rint [70].

Limitations and future developments. The limitations observed in this study occurred in the cathode electrode, which was made of zinc (metallic material) and showed corrosion due to the environment in the last days of operation. Other researchers have also observed this limitation, making it a point for improvement [71]. Excellent performance of the cathode electrode is essential because it is responsible for the reduction kinetics, which are limited by the activation energy barrier, and by not exceeding it due to failures in the electrode, oxidation will not occur efficiently [72]. The activation barrier can be improved by decreasing the activation barrier values with increasing temperature, oxidant concentration, or electrode interface area [73]. Likewise, a limitation was observed in the reflux system used because when using contained agricultural water, the pumps used were clogged with tiny sand particles, generating problems in recirculation.

Bioremediation using MFCs has begun to be called bioelectroremediation, attracting many researchers due to its great potential to obtain a series of benefits in a single experiment [74]. For future developments, individual microorganisms must be used to accelerate the production of electrical energy and bioremediation so that in the future, a consortium of microorganisms that have shown the best results can be used and, in this way, obtain a more efficient MFC than the currently displayed ones [75]. Likewise, the coating of the materials used to manufacture the electrodes is an issue that is gaining importance since electrodes with high electrical conductivity are manufactured from fruit or vegetable waste. The generation of lipids and adsorption of rare earth elements (Ge^3+^, Gd^3+^, and La^3+^) have recently been developed; it has been shown that the use of microalgae in double-chamber MFCs has been performed with great success, but the use of these microalgae to generate sources of electric current, lipids, and for the absorption of rare earth elements may show a new path for MFCs [76].

## 3. Materials and Methods

### 3.1. Manufacturing MFC-SC

**Figure 5:**
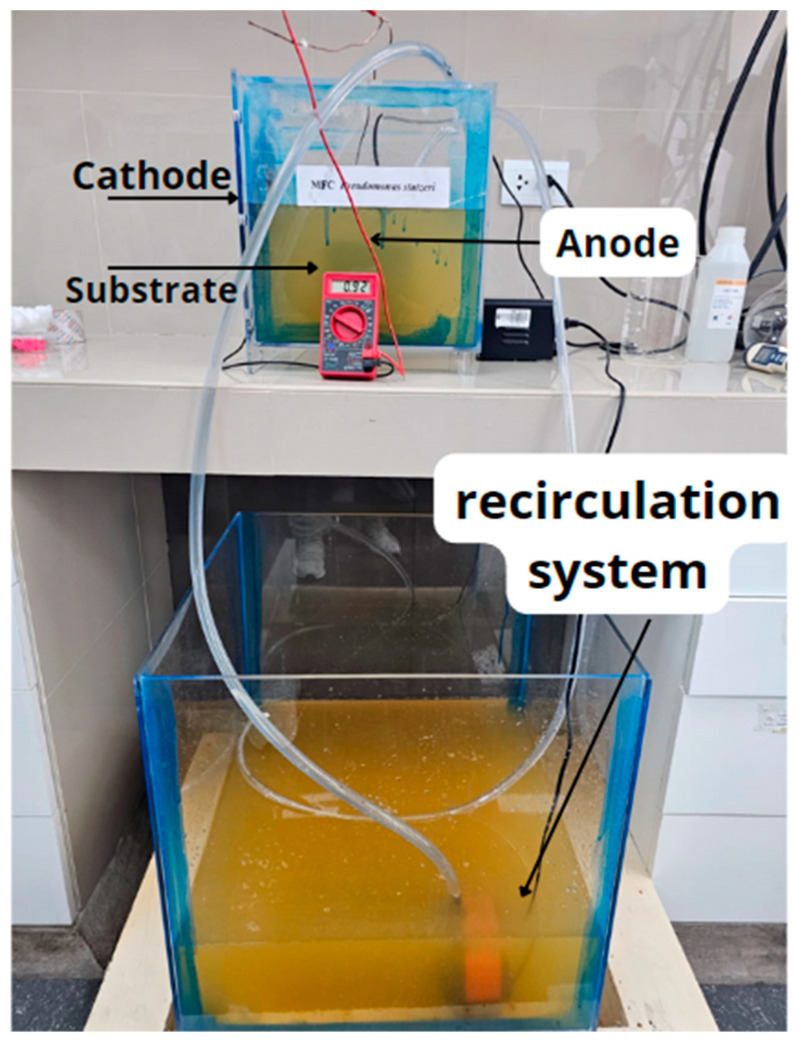
Schematic diagram of single-chamber fuel cells, with recirculation system.

The MFCs used for this investigation were manufactured using polymethylmethacrylate in the form of a prism with 250 cm^3^ capacity, in which a circular orifice was made to place a zinc plate (cathode electrode) of 15 cm radius, while in the center of the MFC a rectangular carbon plate of 200 cm^2^ was placed as an anode electrode. The electrodes were connected externally with a R_ext_ of 100 Ω and internally with a Nafion 117 thin cap, which was used as a PEM to separate both chambers. In addition, two submersible pumps (12 W, Homvik, FL, USA) were placed in the system to act as the recirculation system, as shown in Figure 5.

### 3.2. Irrigation Water Used as Substrate

The irrigation waters used were collected from the Samme irrigation fields, which are fed by the Moche River, Trujillo, La Libertad, Peru. In total, 150 L of this water was collected, which has been contaminated by mining that occurs in the Sierra de Libertad. 

### 3.3. Characterization of Contaminated Waters

The collected data on the substrate in the MFC-SC included the values of total organic carbon (TOC), pH, electrical conductivity (EC), total nitrogen (TN), turbidity, loss on ignition (LOI), dissolved organic carbon (DOC), and chemical oxygen demand (COD), for which a digital multiparameter and turbidimeter were used. The measurements made of total organic carbon, chemical oxygen consumption, dissolved organic carbon, loss of ignition, and nitrogen were obtained through external laboratory contracting, which followed the standard methods used in residual waters. Using the spectrometry of atomic absorption for the remediation of Fe, Cu, and As concentrations, the reduction percentage was found through the formula used in ref. [77].

### 3.4. Obtaining Electrochemical Parameters

The power density and current density values were calculated using the method described by Rojas et al. (2023), where the values of R_ext_ were 0.3 (±0.1), 3 (±0.6), 10 (±1.3), 50 (±8.7), 100 (±9.3), 220 (±13), 460 (±23.1), 531 (±26.8), 700 (±40.5), and 1000 (±50.6) Ω [78]. For the values of the electrical power and electricity, a digital multimeter (Truper MUT—830 Digital Multimeter, Truper, Chihuahua, Mexico) was used whose external resistance was 100 Ω, and the internal resistance was calculated using Ohm’s law.

### 3.5. Molecular Identification of the Bacillus Marisflavi Bacteria

The molecular characterization of sterile cultures of *Bacillus marisflavi* was carried out by Ecobiotechnology S.A.C. The molecular identification process was carried out as recommended by Rojas et al. (2021) based on the analysis of bacterium-specific 16S rRNA [79]. When analyzed with the BLAST program, the percentage of identity was 100%, corresponding to *Bacillus marisflavi* (Table 3).

### 3.6. Bacterial Inoculum

The bacterial inoculum of *Bacillus marisflavi* was obtained from an axenic culture after 22 to 26 h of incubation (plates incubated at 37 °C), suspending the culture in sterile physiological saline solution (SPSS) until obtaining a turbidity similar to MacFarland tube No. 2 (3 × 10^8^ cells/mL). Then, 10 mL of the bacterial suspension was taken and placed in a flask with 90 mL of sterile Trypticase Soy Broth and incubated at 37 °C at 120 rpm for 24 h to obtain a homogeneous liquid bacterial culture, with which the absorbance was measured at 660 nm using a Jenway brand spectrophotometer (Antylia Scientific, Vernon Hills, IL, USA).

### 3.7. Treatment Modules

The treatment modules included a water tank, an MFC, and two water recirculation pumps. The water reservoir was filled with 120 L of sterile contaminated irrigation water (which represents 50% of the reservoir capacity; to sterilize the irrigation water, a calibrated LS-50HD Vertical Pressure Steam Sterilizer (Medwish, Cleveland, OH, USA) with calibration equipment was used; Certificate LCT-A-665-2023) to begin recirculating the water from the reservoir into the MFC and vice versa. This process achieved a flow balance of approximately 3 to 5 L/s. The electrode was then placed inside the MFC; at this point, the first measurements of the bio-electrochemical parameters (voltage, current, conductivity, power density, current density, pH, etc.) were made. After 2 h of the system operating without leaks or imbalances, the bacterial inoculum was added in an amount proportional to 10% of the volume of water to be treated.

## 4. Conclusions

Bioelectricity was generated as expected, and the concentrations of As, Fe, and Cu were successfully reduced using microbial fuel cells with *Bacillus marisflavi* as a biocatalyst. Electric potential and current peaks of 798 mV and 3.519 mA, respectively, were generated, where the MFC–*Bacillus marisflavi* showed an EC of 198.72 mS/cm operating at an optimal pH of 6.54. This MFC reduced the amounts of the toxic metal ions As, Cu, and Fe by 99.08, 56.08, and 91.39%, respectively, in 72 h. Thus, reducing the TN, organic carbon, LOI, DOC, and COD was also possible, with decreases of 69.047, 86.922, 85.378, 88.458, and 90.771%, respectively. At the same time, the internal resistance shown in this investigation was 42.550 ± 12.353 Ω, with a maximum PD of 376.20 ± 15.478 mW/cm^2^ at a CD of 5.713 A/cm^2^. Based on the evaluation of the final results, the treated water can be used without any danger for the irrigation of vegetables because the analyzed parameters are below the maximum permissible limit (MPL) established in the Supreme Decree No. 004-2017-MINAM, where it is established that the MPLs of As and Fe are less than 0.1 and 5.0 mg/kg, respectively, and the MPL of COD is less than 40 mg/L. It is important to respect the values established in current standards given that toxic metal ions in water cause poisoning and deterioration of the health of living beings.

In subsequent research, it is advised to standardize the distance between the electrodes to optimize the power density and manufacture electrodes with nanoparticles capable of more efficiently collecting the electrodes released in the degradation process of organic matter. All this is to give companies and governments a technology capable of generating electrical energy and reducing contaminants in agricultural waters.

## Figures and Tables

**Table 1:** Report of chemical concentrations of MFCs.

		Initial		Final
Parameters	Units		Target	*Bacillus marisflavi*
TN	(mg/L)	1.68	0.56	0.52
TOC	(mg/L)	383.1	76.9	50.1
LOI	(mg/L)	114.9	23.1	16.8
DOC	(mg/L)	245.2	49.2	28.3
COD	(mgO_2_/L)	416.1	56.3	38.4

**Table 2:** Report of the concentrations of Fe, As, and Cu reduced in the bioremediation process.

	Initial (mg/Kg)	Target (mg/Kg)		*Bacillus marisflavi* (mg/Kg)	
	0 h	24 h	72 h	24 h	72 h
Iron	15.09	6.374	2.32	4.798	1.30
Arsenic	0.360	0.210	<0.0033	0.145	<0.0033
Copper	2.12	2.011	1.812	1.352	0.931

**Table 3:** Species identified from marine sediment.

Sample Identification	BLAST Characterization	Length of Consensus Sequence (nt)	% Maximum Identity	Accession Number
**M15**	*Bacillus marisflavi*	1482	100.00	NR_025240.1

## Data Availability

Data are contained within the article.
